# Diffusion-Controlled Release of Bromelain from κ-Carrageenan Nanocomposite Hydrogels Reinforced with Bio-Derived Nanofillers

**DOI:** 10.3390/ijms262311438

**Published:** 2025-11-26

**Authors:** Marisa Faria, Deepa Bhanumathyamma, Gladys Maria Reji, Aswin Sreenivas Baluseri Kuttiyatt, Ghanashyam Sivaprasad, Shanthi Prabha Viswanathan, Artur Ferreira, Jiya Jose, Sreekala Meyyarappallil Sadasivan, Laly Aley Pothan, Nereida Cordeiro, Sabu Thomas

**Affiliations:** 1 LB3, Faculty of Science and Engineering, University of Madeira, 9020-105 Funchal, Portugal; marisa.faria@staff.uma.pt; 2 CIIMAR—Interdisciplinary Centre of Marine and Environmental Research, University of Porto, 4150-196 Porto, Portugal; 3 School of Nanoscience and Nanotechnology, Mahatma Gandhi University, Kottayam 686560, Kerala, India; deepabkaleeckal@gmail.com (D.B.); gladysmariareji@gmail.com (G.M.R.); aswinsreenivasbk@gmail.com (A.S.B.K.); ghanashyamsponniam@gmail.com (G.S.); sreekalams@yahoo.co.in (S.M.S.); lapothan@gmail.com (L.A.P.); 4 IIUCNN—International and Inter University Centre for Nanoscience and Nanotechnology, School of Energy Materials, and School of Nanoscience and Nanotechnology, School of Chemical Science, Mahatma Gandhi University, Kottayam 686560, Kerala, India; 5 ACESSD—Advanced Centre of Environmental Studies and Sustainable Development, Kottayam 686560, Kerala, India; shanthiprabhav@gmail.com; 6 CICECO—Aveiro Institute of Materials, Águeda School of Technology and Management, University of Aveiro, 3754-909 Águeda, Portugal; artur.ferreira@ua.pt; 7 Division of Microbiology, Department of Biosciences, Rajagiri College of Social Sciences (Autonomous), Cochin 683104, Kerala, India; jiyajose@rajagiri.edu

**Keywords:** κ-carrageenan hydrogel, bromelain, cellulose nanocrystals, chitin nanowhiskers, enzyme release, microstructure, antibacterial activity, cytocompatibility

## Abstract

Biopolymer hydrogels are attractive matrices for localised enzyme and drug delivery owing to their intrinsic biocompatibility, biodegradability, and controlled release capacity. In this study, κ-carrageenan hydrogels were engineered as enzyme-delivery systems by reinforcing the matrix with cellulose nanocrystals (CNC) or chitin nanowhiskers (ChNW) and loading bromelain as a model enzyme. The objective was to evaluate how nanofiller chemistry and morphology influence network structure and release behaviour. Parallel fabrication under identical conditions enabled a direct CNC-ChNW comparison. CNC reinforcement compacted the network and reduced swelling, whereas ChNW produced more hydrated and open architectures. Both fillers enhanced surface wettability, while their concentration modulated bulk hydration and diffusivity. Bromelain release over 24 h followed diffusion-controlled kinetics, tunable by filler type and loading. Quantitative topography and pore-size mapping supported structure–function correlations between morphology and transport. All hydrogels were bio-based, biodegradable, and fully cytocompatible, highlighting their suitability for sustainable biomedical applications. Overall, this work provides a quantitative structure-property-function framework for designing enzyme-active κ-carrageenan systems for tunable bromelain release and related biomedical applications.

## 1. Introduction

Hydrogels are hydrophilic three-dimensional polymer networks that retain large amounts of water while maintaining structural integrity. Their inherent softness, porosity, and similarity to the extracellular matrix make them ideal candidates for a range of biomedical applications, including tissue engineering, drug delivery, and wound dressings [1,2]. In addition, their performance is best understood in terms of structure-property-function relationships. Their permeability, quasi-organised water structuring, and diffusion capacity are strongly influenced by ions, proteins, and enzymes present in biological environments, which can modulate swelling and degradation behaviour [3,4,5,6]. When loaded with therapeutic molecules or biologically active agents, hydrogels can act as localised delivery platforms, enabling sustained and controllable release while minimising systemic exposure [7,8]. In this context, there has been growing interest in naturally derived polymers for hydrogel fabrication, due to their biocompatibility, biodegradability, and alignment with principles of green chemistry. Among naturally derived polymers, κ-carrageenan, a sulphated polysaccharide extracted from red algae, has emerged as a particularly attractive base material due to its ion-responsive gelation, biocompatibility, and environmental sustainability. κ-Carrageenan-based hydrogels have been investigated as scaffolds for tissue regeneration, drug carriers, and wound-relevant dressings, in which gelation can be triggered and modulated by physiologically relevant ions [9,10]. In these applications, κ-carrageenan is typically used at concentrations of approximately 1–3 wt% for soft, highly hydrated matrices and 2–4 wt% for mechanically stronger, self-supporting films and dressings [11,12]. Its ability to form thermoreversible and ionotropic gels in the presence of monovalent or divalent cations (e.g., K^+^, Ca^2+^) enables tuning of network rigidity and viscoelastic response. The literature reports that storage moduli can span several orders of magnitude, depending on factors such as polymer concentration, ionic strength, and the type of crosslinker used [11,12]. In addition to its gelling capacity, carrageenan has been explored in composite systems with bacterial cellulose, chitosan, or starch to enhance stability, swelling, or controlled drug release [11,13,14,15,16]. However, despite this versatility, few studies have quantitatively linked the carrageenan network microstructure to functional outcomes such as enzyme release kinetics, antibacterial efficacy, or biodegradation behaviour, parameters that ultimately determine its performance in biomedical applications.

To further enhance the therapeutic function of κ-carrageenan-based hydrogels, the incorporation of bromelain, a proteolytic enzyme complex extracted from pineapple (*Ananas comosus*), has been explored. Bromelain exhibits broad-spectrum bioactivity, including anti-inflammatory, antimicrobial, antiedematous, and wound-debriding effects [16,17]. However, its clinical utility is often limited by susceptibility to degradation under physiological conditions. Encapsulating bromelain within hydrogels offers a strategy for localised and sustained delivery, with more predictable outcomes, while preserving its enzymatic function and maximising therapeutic efficacy [18,19,20]. This combination of clinically relevant bioactivities and the need for controlled local delivery makes bromelain a desirable candidate for incorporation into enzyme-active, wound-relevant hydrogels. Although various hydrogel systems (e.g., alginate, polyethylene glycol (PEG), poly(vinyl alcohol) (PVA)) have been used to deliver bromelain, relatively few studies have systematically examined how nanoscale architecture influences enzyme retention and release.

In addition to serving as simple fillers, biogenic nanofillers such as cellulose nanocrystals (CNC) and chitin nanowhiskers (ChNW) provide quantifiable benefits in polysaccharide hydrogels. Depending on their loading and dispersion, they can increase equilibrium swelling by several tens of percent, modify water uptake and accessible porosity, and adjust effective mesh size and tortuosity, thereby tuning permeability, microstructural organisation, and diffusion-controlled release kinetics of encapsulated solutes [21,22]. In other reported systems, CNC and ChNW have also been associated with increased mechanical stiffness of the hydrogel network, although the magnitude of this effect varies across formulations and was not quantified in the present work [23]. CNC and ChNW, representative forms of nanocellulose and nanochitin, respectively, combine high surface area, intrinsic stiffness or flexibility, and favourable cell-material interactions, making them attractive tools for nanostructural tuning of κ-carrageenan-based hydrogels. Nanocellulose, derived from plant biomass such as pineapple leaves or okara (soybean residue), exhibits high crystallinity and stiffness [24,25,26,27]. Nanochitin, typically derived from crustacean shells or fungi, is highly hydrophilic and biodegradable and, in certain forms, exhibits additional antibacterial properties [28,29]. Both nanomaterials can be sustainably sourced from agro-industrial waste, aligning with circular economy principles, yet differ distinctly in chemistry and morphology: nanocellulose is rigid, hydrophilic, and tends to compact polymer networks, whereas nanochitin is more hydrated, flexible, and potentially bioactive. In combination with κ-carrageenan and bromelain, this complementarity provides a tunable design space in which the balance among network compactness, hydration, wettability, and enzyme release can be finely adjusted, making CNC and ChNW reinforced κ-carrageenan/bromelain hydrogels particularly promising for sustainable, wound-relevant delivery systems. Taken together, these features motivate a direct, mechanistically oriented comparison of CNC and ChNW reinforced κ-carrageenan/bromelain hydrogels to clarify how nanofiller identity governs structure-function relationships. Moreover, the combination of κ-carrageenan and bromelain has been only superficially explored in the literature, despite their potential synergistic effects for wound dressings and enzymatic debridement [18,19,20]. One plausible explanation for the relatively limited exploration of bromelain-based delivery systems is their niche application, where they compete with other established protease-based dressings and carrier matrices, thereby reducing the perceived need for alternative platforms. To our knowledge, no study has provided a standardised, head-to-head comparison of nanocellulose vs. nanochitin within the same κ-carrageenan/bromelain system under identical processing conditions, using unified fabrication and characterisation protocols. This lack of standardisation has hindered a comprehensive understanding of nanofiller contributions to the structural and functional performance of bioactive hydrogels. A quantitative understanding of how nanofiller chemistry and topology straightforwardly influence pore morphology, surface roughness, and subsequent functional outputs (enzyme release, cytocompatibility, biodegradation) would enable predictive material design, moving from descriptive formulation studies toward data-driven biomaterial engineering.

**Figure 1:**
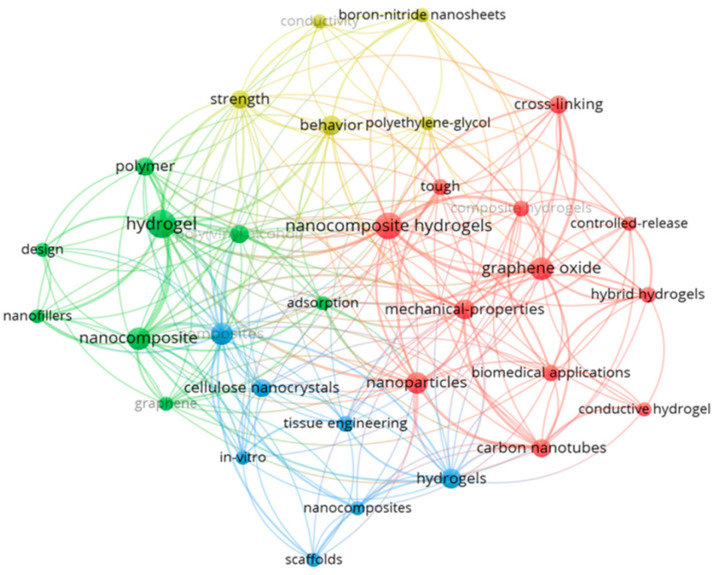
Bibliometric co-occurrence map of keywords extracted from scientific publications indexed in the Web of Science [30] using the query “hydrogel AND polymer AND nanofillers AND biomedical”. The analysis was performed using VOSviewer software (version 1.6.20) [31]. Each node represents a keyword, with node size proportional to its frequency of occurrence, and link thickness corresponding to the strength of co-occurrence with other terms. The map is organized into distinct clusters identified by color: the red cluster is centered on “nanocomposite hydrogels,” “graphene oxide,” and related biomedical and mechanical performance themes; the green cluster focuses on foundational terms like “hydrogel,” “polymer,” and “nanocomposite”; the blue cluster highlights biomedical engineering aspects such as “scaffolds,” “tissue engineering,” and “cellulose nanocrystals”; and the yellow cluster connects terms such as “strength,” “conductivity” and “boron-nitride nanosheets,” indicating performance enhancement research.

In this context, the bibliometric co-occurrence map (Figure 1) is used in a strictly descriptive manner to illustrate how rarely the specific combination of “hydrogel”, “κ-carrageenan”, “bromelain”, and nanocellulose/nanochitin-related terms appears in the indexed literature. The map shows three dominant clusters related to (i) generic polymeric hydrogels and wound-healing terms, (ii) nanocellulose/nanochitin and nanocomposites, and (iii) enzyme-loaded systems and drug delivery. Explicit co-occurrence of terms such as ‘bromelain’, ‘κ-carrageenan-bromelain’, or ‘bromelain hydrogel’ is scarce, which is consistent with the niche character of these systems and the presence of competing formulation strategies, but does not, by itself, provide mechanistic or translational insight. Explicit co-occurrence of terms such as ‘bromelain’, ‘κ-carrageenan-bromelain’, or ‘bro-melain hydrogel’ is scarce, consistent with the niche character of these systems and the presence of competing formulation strategies. However, this does not provide mechanistic or translational insights. Accordingly, Figure 1 is used solely to contextualise the under-representation of κ-carrageenan/bromelain nanocomposites at the intersection of these research areas and to motivate the present standardised comparison, rather than to support broader conclusions about clinical relevance or adoption.

Despite multiple studies describing κ-carrageenan composites reinforced with nanocellulose, chitin, or other biogenic fillers, most reports differ in processing variables, polymer concentration, or crosslinking conditions, which complicates direct comparison. Furthermore, systematic quantification of how nanofiller chemistry and morphology control network topology and diffusive transport remains limited. There is a lack of standardised, head-to-head analyses performed under identical synthesis conditions. This study addresses that gap by directly comparing CNC and ChNW within the κ-carrageenan-bromelain system, establishing quantitative links between microstructural descriptors (pore size, roughness, wettability) and functional responses (hydration, enzyme release, antibacterial activity, and cytocompatibility). The central aim is to generate a standardised, quantitative, head-to-head comparison of CNC and ChNW reinforced κ-carrageenan/bromelain hydrogels under identical processing conditions, thereby mapping structure-microstructure-response relationships that can guide rational formulation design.

This work establishes a proof-of-concept by developing two κ-carrageenan/bromelain hydrogel systems, reinforced with either CNC or ChNW nanofillers, prepared under identical processing conditions. Both nanomaterials were extracted using sustainable green protocols from waste biomass, and the hydrogels were fabricated via a facile, reproducible synthesis approach. A series of physicochemical and biological characterisations were performed to assess and compare swelling behaviour, surface wettability, bromelain release kinetics, biodegradation profile, and cytocompatibility under controlled conditions. This integrated physicochemical and biological characterisation, including quantitative pore-size and roughness analysis (scanning electron microscopy (SEM) and atomic force microscopy (AFM)), enzyme-release kinetics, antibacterial profiling, biodegradation, and cytocompatibility assays, provides a direct, data-correlated link between microstructure and function. The resulting evidence supports the creation of a reproducible, sustainable design map for enzyme-active hydrogels and offers generalizable insights applicable to other natural polymer-nanofiller systems for wound care and drug delivery.

## 2. Results and Discussion

### 2.1. Effects of Nanofillers on Network Architecture (CNC vs. ChNW)

#### 2.1.1. Macroscopic Integrity and Chemical Resilience

**Figure 2:**
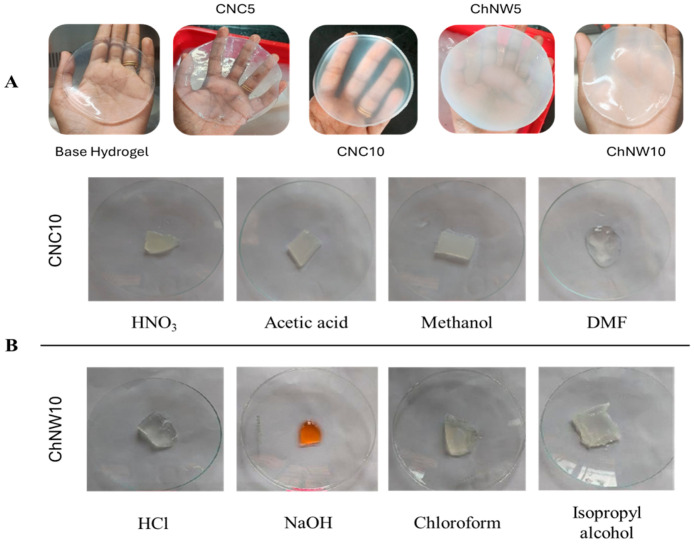
Macroscopic appearance and short-term chemical challenge of κ-carrageenan/bromelain hydrogels. (**A**) base hydrogel (no nanofiller), CNC5, CNC10, ChNW5, and ChNW10 immediately after casting and ZnCl_2_-mediated ionotropic crosslinking (1 wt%, 30 min; disc diameter = 11.0 cm). (**B**) Representative solvent challenge after 2 h at 22 ± 2 °C. Top (CNC10): diluted HNO_3_, acetic acid, methanol, DMF. Bottom (ChNW10): 0.1 N HCl, 0.1 N NaOH, chloroform, isopropyl alcohol (glass plate diameter 9 cm).

Macroscopic evaluation revealed systematic differences in transparency, cohesion, and handling as a function of nanofiller type and loading. All formulations formed stable matrices without visible phase separation, confirming gel formation of the κ-carrageenan/bromelain system. The base hydrogel displayed the greatest optical clarity. CNC reinforced discs remained largely transparent; CNC10 appeared more transparent and qualitatively more rigid (less prone to deformation) during handling than CNC5 and the base hydrogel. This observation aligns with its visually denser network, although no quantitative mechanical tests were conducted in this study (Figure 2A). These trends are consistent with nanofiller-dependent network architectures reported for polysaccharide hydrogels.

A 2 h solvent challenge at 22 ± 2 °C indicated solvent-dependent resilience (Figure 2B). CNC10 retained gross structure and clarity in diluted HNO_3_, acetic acid, and methanol, but showed loss of integrity in DMF. ChNW10 remained intact in HCl, chloroform, and isopropyl alcohol; exposure to NaOH produced marked orange discolouration and erosion/shrinkage. In some samples, isopropyl alcohol yielded a firmer appearance, consistent with solvent-induced dehydration. These qualitative observations delineate practical handling windows (stable in dilute acids and protic alcohols; vulnerable in strong bases and DMF) [24,29,32].

#### 2.1.2. Fourier–Transform Infrared Spectroscopy (FTIR) Analysis

**Figure 3:**
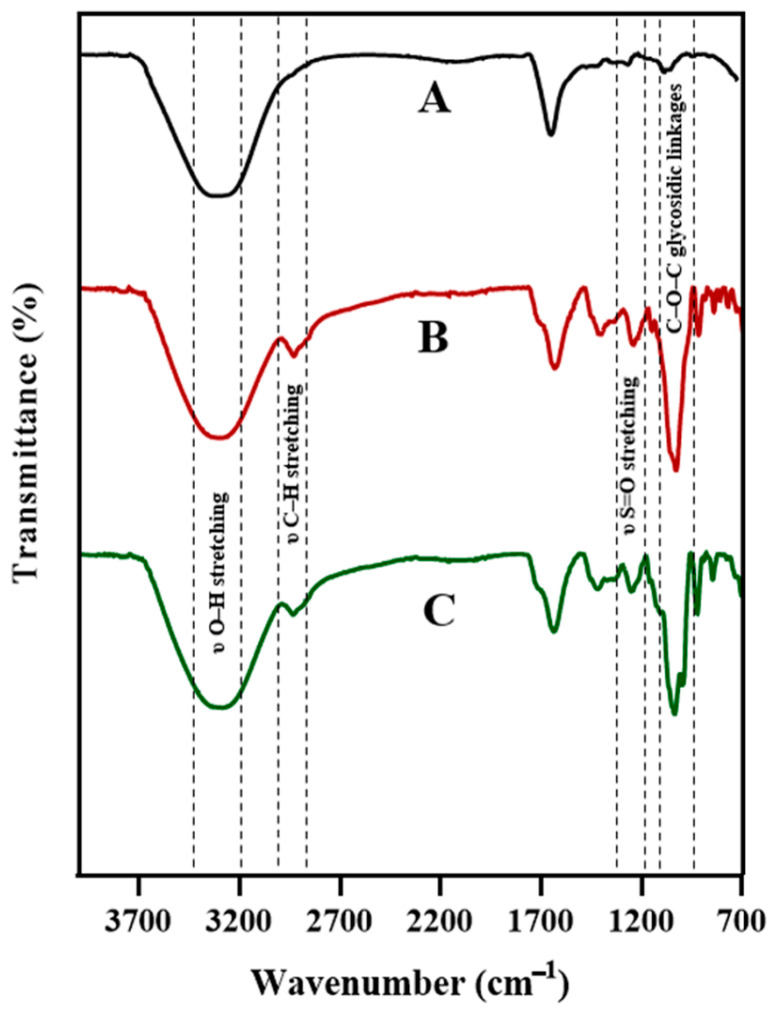
FTIR spectra of base hydrogel (black line; A), CNC10 (red line; B), and ChNW10 (green line; C). Characteristic polysaccharide and nanofiller bands, as well as changes upon bromelain loading, are highlighted.

FTIR was used to probe chemical signatures and interactions in the base and composite hydrogels (CNC10, ChNW10). All spectra (Figure 3) displayed a broad O–H stretching band at 3200–3400 cm^−1^, consistent with hydrophilic polysaccharide matrices, and the 1220 cm^−1^ sulfate-ester (S=O) stretching characteristic of κ-carrageenan [10]. Bands near 1030 cm^−1^ correspond to C–O–C glycosidic linkages. In κ-carrageenan/CNC/bromelain, an intensified 2900 cm^−1^ C–H stretching band is consistent with cellulose incorporation. In κ-carrageenan/ChNW/bromelain, a band near 1550 cm^−1^ (amide II; N–H bending of acetamido groups) is consistent with chitin [28,29]. Subtle shifts and band broadening upon bromelain loading are consistent with non-covalent interactions (e.g., hydrogen bonding) between the enzyme and the polymer network [18].

#### 2.1.3. AFM Topography

**Figure 4:**
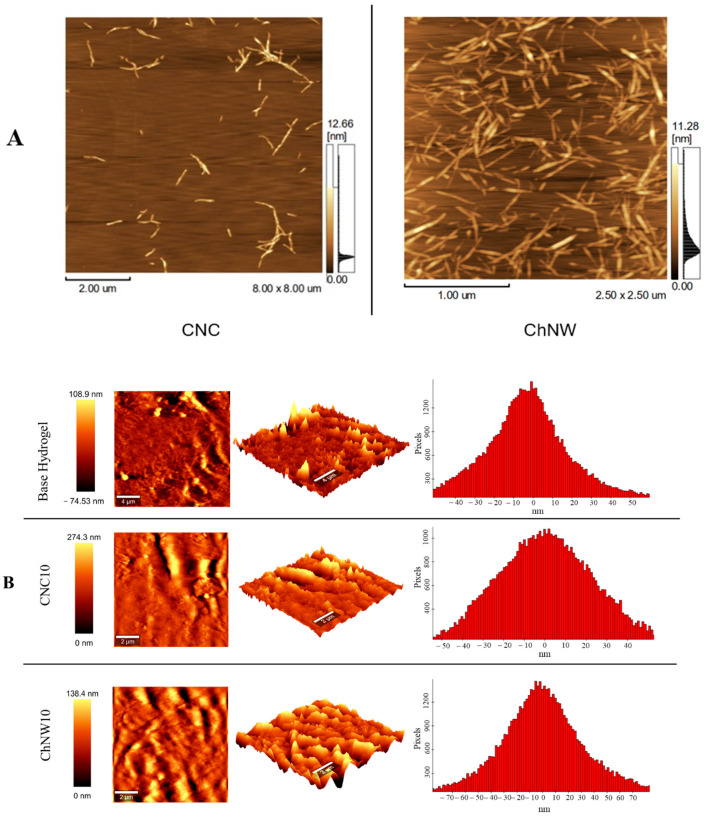
AFM analysis of nanofillers and κ-carrageenan/bromelain hydrogel surfaces. (**A**) AFM height images of the extracted nanomaterials: cellulose nanocrystals (CNCs) and chitin nanowhiskers (ChNWs). (**B**) AFM topography of the base hydrogel, CNC10, and ChNW10. Left: 2D height maps; middle: 3D renders; right: height histograms.

AFM of the extracted nanomaterials confirmed the expected morphologies (Figure 4A). CNC exhibited a short rod-like (needle-like) shape with an average length of 430.04 ± 144.70 nm and a width of 5.63 ± 1.77 nm. ChNW displayed a nanowhisker-like morphology with an average length of 328.56 ± 101.08 nm and a width of 3.22 ± 1.44 nm. These dimensions are consistent with those of CNC and ChNW produced by acid hydrolysis, supporting the successful preparation of these materials before incorporation into the hydrogels [21,33].

AFM of the hydrogel surfaces (Figure 4B) revealed distinct morphologies, strongly influenced by the type and content of nanofillers. Compared with the base hydrogel, both composites exhibited higher surface roughness and expanded height ranges. CNC10 displayed sharper asperities and a more tightly packed texture, whereas ChNW10 presented more open, undulating domains. These topographical differences are expected to propagate to macroscopic behaviour: CNC induced compaction should lower swelling and restrict diffusive transport, whereas ChNW induced hydrated/open domains should increase swelling and permit greater fluid ingress; consequently, distinct enzyme-release profiles are anticipated, with denser networks releasing more slowly.

Quantitative surface roughness parameters obtained from AFM height maps (5 µm × 5 µm) confirmed these trends. The base hydrogel exhibited $S_{a}$ = 22.15 nm and $S_{q}$ = 31.84 nm, CNC10 showed $S_{a}$ = 22.92 nm and $S_{q}$ = 29.22 nm; ChNW10 displayed $S_{a}$ = 33.53 nm and $S_{q}$ = 47.52 nm. The increased roughness in ChNW10 reflects the formation of hydrated, topographically heterogeneous domains, consistent with its enhanced swelling behaviour. The slight rise in CNC10 relative to the base hydrogel suggests a more compact, topographically rigid structure, consistent with its comparatively compact architecture.

Together, these data demonstrate that nanofiller chemistry and morphology govern nanoscale surface architecture, which in turn modulates hydration, wettability, and bromelain release.

#### 2.1.4. Field-Emission Scanning Electron Microscopy (FE–SEM) Microstructure

**Figure 5:**
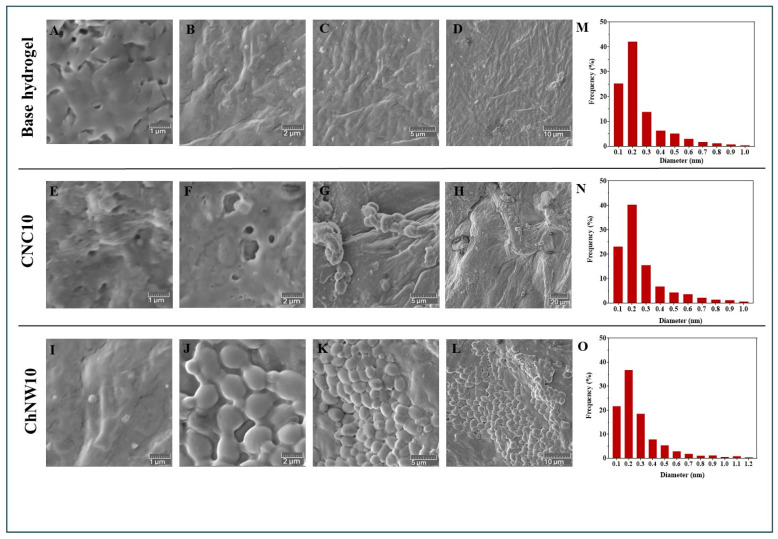
FE–SEM micrographs of κ-carrageenan/bromelain hydrogels at multiple magnifications. (**A**–**D**) base hydrogel; (**E**–**H**) κ-carrageenan-CNC-bromelain hydrogel (10%, CNC10); (**I**–**L**) κ-carrageenan-ChNW-bromelain hydrogel (10%, ChNW10). Histograms show the corresponding pore size distributions, calculated from SEM images using ImageJ (**M**–**O**): pore-size distributions and field porosity (%) calculated from ImageJ (**M**–**O**).

FE-SEM micrographs (Figure 5A–D) showed a dense, relatively smooth surface for the base hydrogel with limited visible pores. Quantitative pore analysis confirmed its compact morphology; the mean pore diameter increased modestly with CNC and more clearly with ChNW (base = 0.26 µm; CNC10 = 0.28 µm; ChNW10 = 0.30 µm), consistent with a transition from a compact to a more open network. In contrast, the nanofiller-reinforced hydrogels exhibited more porous and heterogeneous microstructures. CNC10 presented a fibrillar or networked pattern (Figure 5E–H) with a mean pore diameter of about 0.28 µm, reflecting local aggregation of CNC that slightly disrupted the κ-carrageenan matrix. ChNW10 displayed a sponge-like texture with a more open, interconnected pore network (Figure 5I–L), yielding the largest mean pore size (0.30 µm). The fibrous ChNW promoted the formation of elongated microchannels, thereby increasing surface porosity and connectivity within the gel matrix. The development of these interconnected microcavities supports enhanced hydration and molecular transport pathways, framing the swelling and release behaviours reported in subsequent sections [28,29].

These morphological differences can be interpreted in terms of effective mesh size and tortuosity. The higher crystallinity and rigidity of CNC promote closer chain packing and denser hydrogen bonding, leading to reduced free volume and lower permeability. In contrast, ChNW, containing acetamide and hydroxyl groups, interacts more weakly with κ-carrageenan helices and retains bound water, forming a hydrated, interconnected network. These structural contrasts are expected to modulate diffusive transport and are consistent with the subsequent release profiles.

Overall, under standardised fabrication, nanofiller selection dictates network architecture: CNC favours optically more transparent, more compact networks, and ChNW favours more hydrated, open networks. This structural basis rationalises subsequent differences in wettability and hydration, as well as in bromelain release and antibacterial performance.

### 2.2. Surface Wettability and Hydration

**Figure 6:**
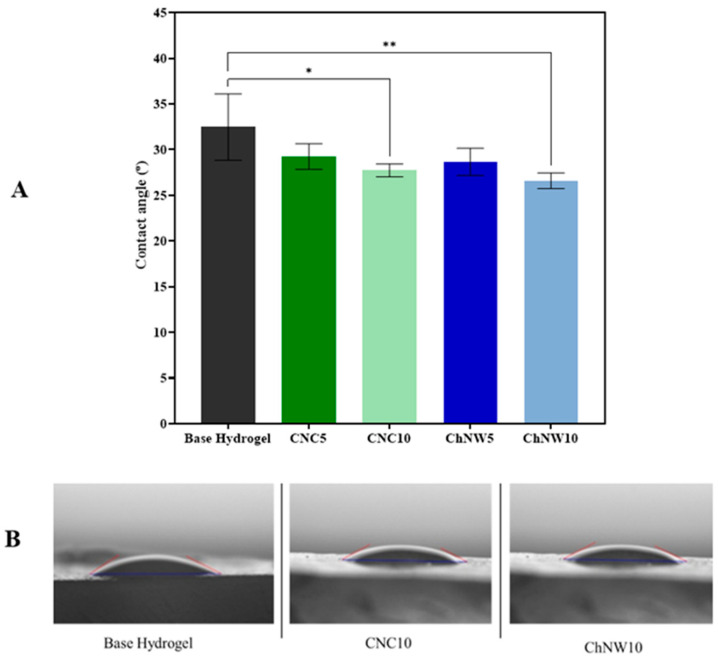
Surface wettability of κ-carrageenan/bromelain hydrogels. (**A**) Static water contact angle (mean ± SD; n = 3). Asterisk denote significant differences compared to the base hydrogel (* *p* < 0.05; ** *p* < 0.01). (**B**) Representative sessile-drop images.

Static contact-angle measurements (Figure 6) indicated that all nanofiller-reinforced formulations were more wettable than the base hydrogel. The base hydrogel showed 32.4°, whereas CNC5 = 29.2°, CNC10 = 27.6°, ChNW5 = 28.6°, and ChNW10 = 26.6°. The small but consistent reductions are attributed to: (i) increased surface polarity from hydroxyl (CNC) and acetamide/hydroxyl (ChNW) groups presented at the interface, and (ii) the roughness increase observed by AFM, which, on intrinsically hydrophilic surfaces (*θ* < 90°), is expected to lower the apparent angle further. The trend aligns with reports that nanocellulose or nanochitin fillers enhance the hydrophilicity of polysaccharide matrices through surface chemistry and interfacial structuring [24,29,34]. In the context of wound care, greater wettability promotes rapid fluid spreading and conformal interfacial contact with the wound bed, improving adherence and mass transfer.

Correlating these results with the quantitative morphological data, lower contact angles coincide with higher $S_{a}/S_{q}$ and larger pore sizes, demonstrating that nanoscale roughness and porosity collectively enhance surface energy and water affinity. The ChNW10 hydrogel, possessing both the highest roughness ($S_{a}$ = 33.5 nm, $S_{q}$ = 47.5 nm) and the largest average pore diameter (0.3 µm), therefore displays the greatest hydrophilicity and hydration capacity.

**Figure 7:**
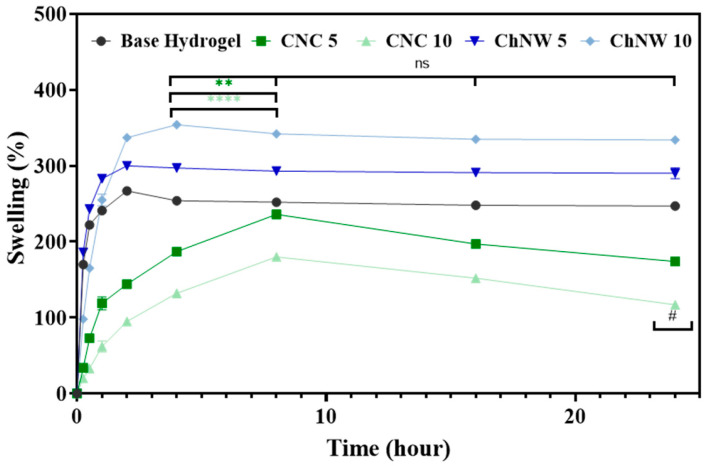
Swelling behaviour of κ-carrageenan-bromelain hydrogels over 24 h in PBS (pH 7.4) at 37 °C. (**#**) indicates statistically significant differences compared to the base hydrogel group. ns = not significant; Asterisk indicate significant differences among hydrogel groups (** *p* < 0.01 and **** *p* < 0.0001).

Swelling in phosphate-buffered saline (PBS; pH 7.4) (Figure 7) increased rapidly at early times and reached a plateau by 4–8 h; changes at 24 h were minor and not statistically significant. At 24 h the ordering was ChNW10 > ChNW5 > base hydrogel > CNC5 > CNC10, with representative magnitudes of 354, 310, 267, 236 and 180%, respectively. Group differences were significant at and beyond 8 h (one-way ANOVA/Tukey): both ChNW formulations were higher than base and CNC (*p* ≤ 0.01), and CNC10 was lower than base and CNC5 (*p* ≤ 0.01). These contrasts are consistent with the nanofiller-dependent architectures established previously: AFM/SEM showed that CNC tends to compact the κ-carrageenan network (tighter packing, sharper asperities), which limits free volume and water uptake at higher loadings; by contrast, ChNW promotes more hydrated/open domains that facilitate fluid intake. Similar behaviours, namely densification with reduced swelling in CNC reinforced systems and increased water uptake in chitin-based networks, have been reported for polysaccharide hydrogels [24,28,29]. A trade-off between network cohesion and swelling capacity is a common feature of hydrogel design [35]. Taken together, wettability and swelling are co-modulated by nanofiller selection and loading: both CNC and ChNW lower the contact angle; however, only ChNW produces a marked increase in bulk hydration over 24 h, whereas higher CNC loading suppresses swelling. This hydration landscape is directly relevant to exudate management and helps rationalise the enzyme-release profiles.

### 2.3. Bromelain Release Profiles from CNC and ChNW Reinforced Hydrogels

**Figure 8:**
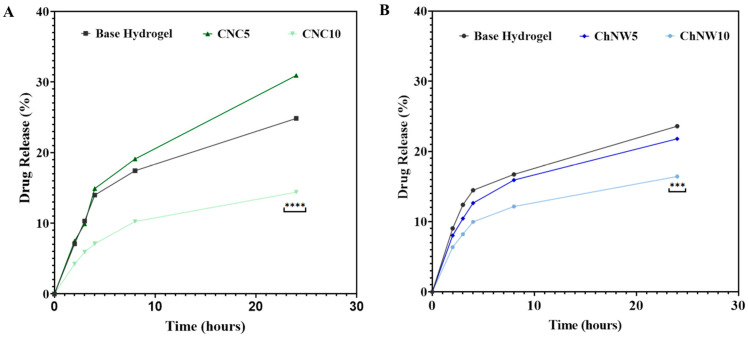
Cumulative bromelain release (%) from κ-carrageenan hydrogels reinforced with CNC or ChNW (5 or 10 wt% relative to κ-carrageenan) in PBS (pH 7.4) at physiological temperature (37 ± 2 °C) under sink conditions. (**A**) CNC series (base hydrogel, CNC5, CNC10). (**B**) ChNW series (base hydrogel, ChNW5, ChNW10). Data are mean ± SD (n = 3). Asterisk indicate significant differences vs. base hydrogel (*** *p* < 0.001, **** *p* < 0.0001).

Bromelain release from the κ-carrageenan hydrogels was evaluated in PBS (pH 7.4) at physiological temperature (37 ± 2 °C) under sink conditions (Figure 8; Table 1). All formulations exhibited time-dependent, progressive release, consistent with diffusion modulated by the network architecture. At 24 h, the cumulative release was ranked as follows: CNC5 (30.9%) > base hydrogel (24.8%) > ChNW5 (21.8%) > ChNW10 (16.4%) > CNC10 (14.4%), differences vs. the base hydrogel are indicated in Figure 8.

Compared with other hydrogel-based delivery systems, the bromelain release profiles obtained here are slower. Several polymeric matrices for bromelain delivery, such as alginate/Arabic gum hydrogels, PVA/PEG hydrogels, and pectin- or alginate-based beads, have been shown to release a substantial fraction of the enzyme within the first few hours of incubation in aqueous buffers or physiological conditions [18,36,37,38]. In many of these systems, cumulative bromelain release over 24 h exceeds 50–70% under sink conditions. In contrast, the κ-carrageenan/CNC and κ-carrageenan/ChNW hydrogels developed in this work released only 14–31% of the enzyme over 24 h (Figure 8). This slower release profile suggests a more sustained, diffusion-limited regime. This behaviour is consistent with the denser, more confining networks observed for CNC10 and with the higher affinity and tortuosity introduced by chitin-rich ChNW domains, and may be advantageous in local therapeutic scenarios where prolonged, low-level exposure is preferred over rapid burst release.

The observed release trends can be directly rationalised by the microstructural and topographical parameters derived from AFM and SEM analyses. CNC10, with its compact architecture, restricts diffusive transport, resulting in the lowest cumulative release (14.4% at 24 h). In contrast, the ChNW10 hydrogel, characterised by enlarged pores and higher roughness, facilitates greater fluid infiltration and enzyme mobility, leading to moderately faster release than CNC10 despite stronger polymer–protein interactions. These quantitative correlations reinforce that surface topology and internal porosity act as tunable design levers for controlled enzyme delivery in κ-carrageenan matrices.

Under matched conditions, lower CNC loading (CNC5) increased 24-h cumulative release above the base hydrogel, whereas higher loading (CNC10) reduced release, consistent with network densification that restricts diffusive transport at elevated CNC content. For ChNW, both loadings resulted in lower cumulative release than the base, even though swelling increased. This suggests that a higher degree of bulk hydration does not automatically lead to quicker solute transport. This observation aligns with the presence of more hydrated, physically cross-linked networks and stronger interactions between the polymer and the enzyme/nanofiller, which slow diffusion. Interestingly, ChNW10 combined the largest pore sizes and the highest swelling ratio with a comparatively low cumulative release. This apparent decoupling suggests that bromelain transport is governed not only by average pore size but also by network tortuosity and specific interactions. The high ChNW content likely increases the effective path length and creates heterogeneous microdomains in which electrostatic and hydrogen-bonding interactions with sulphated carrageenan and chitin surfaces transiently immobilise bromelain. In addition, the more hydrated, gel-like ChNW rich domains may favour bound water layers and ‘pore shielding’, slowing convective exchange despite high overall water uptake. Similar behaviour, where highly swollen polysaccharide networks still exhibit slowed protein release due to strong matrix affinity and tortuous diffusion pathways, has been reported for hydrogels containing charged nanofillers [21,25,26,29].

Release proceeds through an early hydration/expansion phase (0–4 h) followed by a diffusion-limited tail (4–24 h) (Table 1). In the first hours, rapid wetting and mesh expansion drive the highest rates; CNC5 and ChNW5 show faster early release owing to more accessible aqueous pathways, whereas the denser CNC10 network starts slower. Within this early window, CNC5 exhibits a transient maximum (around 3–4 h), consistent with pore percolation. Beyond 4–8 h, the mobile fraction is depleted, the network stabilises, and path length/tortuosity increases; rates drop to ≤1%·h^−1^ and remain uniformly low through 24 h.

Fitting the release data (up to 24 h, corresponding to ≤30% cumulative release) to the Higuchi model produced linear correlations (R^2^ > 0.95) for all formulations, confirming diffusion-controlled transport within hydrated networks. The apparent Higuchi constants decreased in the order CNC5 > base hydrogel > ChNW5 > ChNW10 > CNC10, indicating that increasing filler content and network compactness reduced effective diffusivity. Complementary fitting to the Korsmeyer-Peppas model yielded release exponents 0.42–0.61, consistent with Fickian to slightly anomalous diffusion. Lower n values for CNC10 reflect restricted molecular mobility in dense matrices, whereas higher values for ChNW10 suggest partial polymer relaxation in more hydrated networks.

Analysis of bromelain release as a function of nanofiller loading revealed a clear concentration-dependent trend. Increasing CNC from 5 to 10 wt% markedly decreased both k_H_ and cumulative release (30.9 to 14.4%), while ChNW systems showed a milder, non-linear dependence (21.8 to 16.4%), reflecting the combined effects of network density and interfacial chemistry.

In practical terms, CNC5 enabled faster but still diffusion-controlled enzyme release, suitable for short-term therapeutic activity, whereas CNC10 and ChNW10 provided more sustained profiles. The interplay between nanofiller type and concentration allows precise tuning of release kinetics, supporting the rational design of bio-based hydrogels with controllable diffusional properties. Such tunability is particularly relevant for enzyme-active wound dressings, where a balance between rapid onset and prolonged action is critical. This controllable diffusion behaviour highlights the potential of these bio-based hydrogels as adaptable matrices for future therapeutic and biotechnological applications.

### 2.4. In Vitro Antibacterial Activity

Antibacterial activity was assessed by disc diffusion against *Staphylococcus aureus* and *Klebsiella pneumoniae*. Free bromelain produced the largest zones of inhibition (set as 100%), whereas the base hydrogel (no bromelain) showed no inhibition. Among bromelain-loaded hydrogels, CNC5 showed the largest ZOI, while the higher loading formulations (CNC10 and ChNW10) showed the smallest; all hydrogels exhibited significantly smaller ZOIs than free enzyme (Table 2). The largest ZOI (CNC5) coincided with the highest 24 h cumulative release (Figure 8), whereas higher-loading formulations (CNC10, ChNW10), which released more slowly, showed the smallest ZOI. Some strain-dependent differences in ranking were noted; overall, the pattern supports a release-limited antibacterial effect under agar diffusion conditions [18].

Quantitative morphological descriptors were consistent with antibacterial efficacy: formulations with rougher, more open microstructures enable broader bacterial contact and the diffusion of released bromelain, whereas overly compact networks limit exposure. Thus, micro- to nanoscale surface architecture governs both enzyme release kinetics and subsequent biological performance.

As expected, the base κ-carrageenan hydrogel without bromelain produced no detectable inhibition zones (0 mm) for either *S. aureus* or *K. pneumoniae*. In contrast, all bromelain-loaded nanocomposite hydrogels showed clear inhibition halos, with diameters summarised in Table 2. Each value in Table 2 corresponds to the mean ± standard deviation of three independently prepared plates per formulation and strain. Independent “nanofiller-only” hydrogels (containing CNC or ChNW but lacking bromelain) were not included in the original experimental design. Consequently, a possible intrinsic antibacterial effect of the nanofillers cannot be fully excluded and should be addressed in future studies by incorporating nanofiller-loaded, bromelain-free control formulations.

### 2.5. Cytocompatibility and Biodegradation

#### 2.5.1. Cytocompatibility (MTT, L929 Fibroblasts)

**Figure 9:**
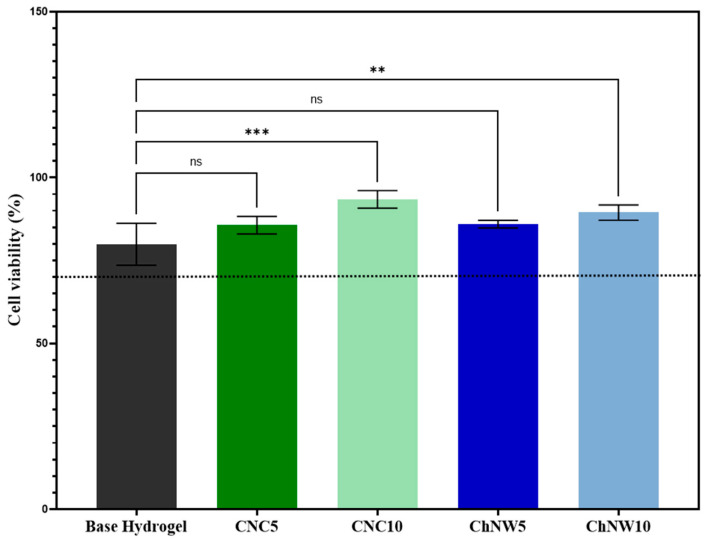
Metabolic activity (cell viability, %) of L929 fibroblasts after 24 h exposure to extracts of κ-carrageenan/bromelain hydrogels (base, CNC5, CNC10, ChNW5, ChNW10), assessed by the MTT assay. Data are expressed as a percentage of untreated control cells (mean ± SD, n = 3; the horizontal dashed line at 70% represents the ISO 10993-5 cytotoxicity threshold for non-cytotoxic materials. Asterisks indicate significant differences vs. base hydrogel (** *p* < 0.01, *** *p* < 0.001); ns = not significant.

All formulations were non-cytotoxic at 24 h (Figure 9), with all conditions ≥ 70% of untreated controls (ISO 10993-5) [39]. The ranking of metabolic activity was CNC10 (93.4%) > ChNW10 (89.5%) > ChNW5 (86.0%) > CNC5 (85.6%) > base hydrogel (79.9%). Values below 100% reflect slightly reduced metabolic activity compared to untreated cells, yet remain within the non-cytotoxic range, while values approaching 100% suggest preserved or enhanced activity. This ordering is consistent with nanofiller-driven interfacial changes, greater surface hydrophilicity/polarity (lower contact angle), and nanoscale topography (AFM), known to support fibroblast adhesion and metabolism in polysaccharide matrices [12,34,40,41]. ChNW, bearing acetamide and hydroxyl groups, plausibly provides a more polar, cell-favouring interface than CNC under these conditions; bromelain may also contribute to a pro-regenerative milieu [42,43]. Overall, the data support the cytocompatibility of both CNC and ChNW reinforced κ-carrageenan/bromelain hydrogels under the in vitro conditions tested, indicating that these formulations are suitable candidates for further evaluation in wound-relevant models.

When set against other bromelain delivery systems, the κ-carrageenan nanocomposites developed here occupy a distinct kinetic regime. Thermoresponsive poly(N-isopropylacrylamide) (PNIPAAm)-based hydrogels, for example, have been reported to release a large fraction of loaded bromelain within the first hours of incubation, reflecting relatively loose network constraints and limited specific interactions with the enzyme. By contrast, the present κ-carrageenan/CNC and κ-carrageenan/ChNW matrices restrict cumulative release to ca. 14–31% over 24 h, indicating a more strongly confining network, in line with the observed smaller pore sizes (CNC) and the higher affinity of chitin-rich domains (ChNW). This slower, diffusion-controlled profile may be advantageous for local applications where prolonged, low-level exposure to enzymatically active bromelain is desired rather than rapid burst release.

#### 2.5.2. Biodegradation Behaviour

**Figure 10:**
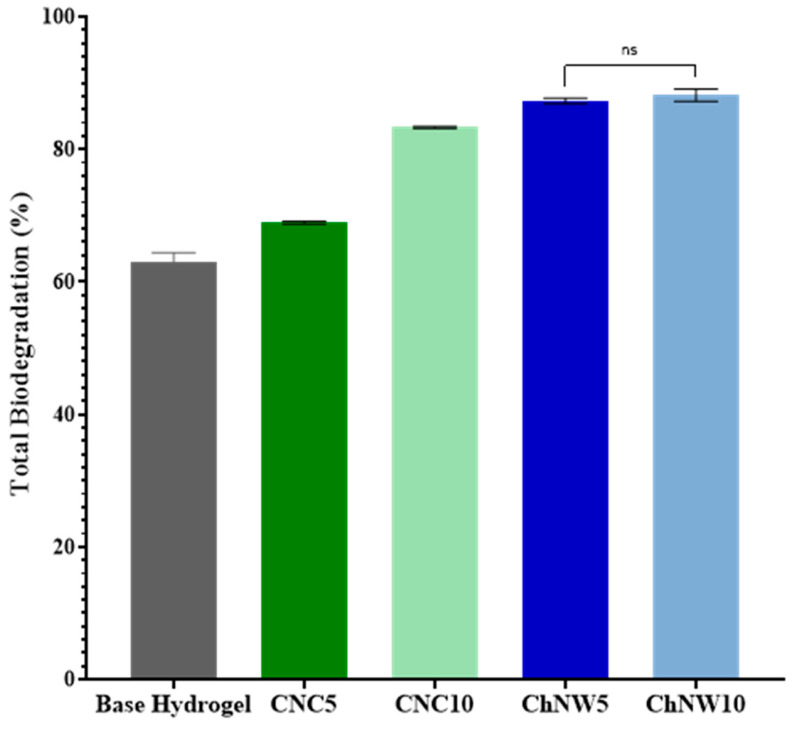
Biodegradation behaviour of κ-carrageenan/bromelain hydrogels (base, CNC5, CNC10, ChNW5, ChNW10) during soil-burial testing over 7 days at 25 ± 2 °C, expressed as mass loss (%) vs. initial dry mass (mean ± SD, n = 3). ns: not significant.

Biodegradability was assessed using a soil-burial assay over 7 days (Figure 10). All formulations exhibited progressive mass loss, indicating environmental responsiveness. On day 7, nanofiller-reinforced hydrogels exhibited greater mass loss than the base hydrogel, with higher loadings giving the largest values; however, differences among the nanofiller groups did not reach statistical significance (*p* ≥ 0.05). The directional increase with nanofiller content is consistent with the structural changes documented earlier, higher surface area/roughness (AFM/SEM), increased wettability (lower contact angle), and more open microstructures, particularly with ChNW, which can facilitate microbial access and enzyme-mediated breakdown. This behaviour aligns with reports that nanocellulose and nanochitin modulate hydrogel degradability by enlarging the accessible surface and promoting microbial colonisation [12,44]. Overall, the materials exhibit a transient profile compatible with single-use wound dressings. However, ChNW based hydrogels showed the highest degradation trend; confirmation of faster breakdown would benefit from longer time points and enzyme-assisted assays (e.g., cellulase, chitinase, lysozyme) to complement the environmental soil burial test.

Taken together with the morphological results, the data suggest that nanofillers influence the overall “lifetime envelope” of the hydrogel: smoother, denser CNC networks (lower S_a_, smaller pores) are more resistant to hydration and microbial erosion, whereas rougher, open ChNW networks (higher S_a_/S_q_, larger pores) favour faster biodegradation and bio-interface turnover which features advantageous for temporary wound-dressing applications.

**Figure 11:**
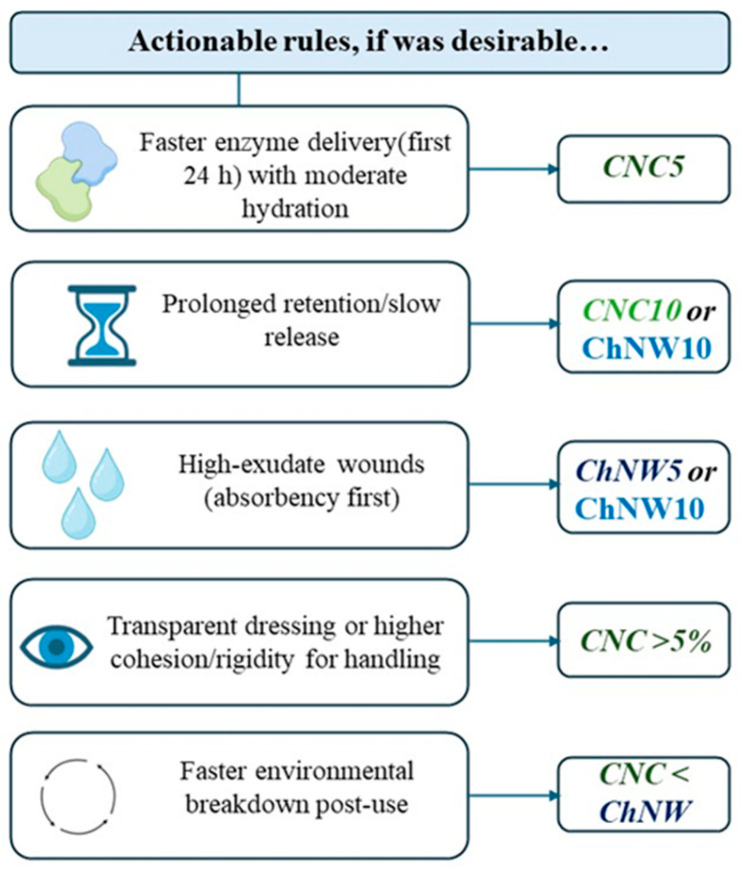
Design map for selecting CNC vs. ChNW in κ-carrageenan/bromelain hydrogels.

Integrating the preceding data and literature, a design map was developed to guide the selection of CNC vs. ChNW for specific use cases. The head-to-head comparisons condense into practical rules summarized in Figure 11: CNC5 for faster 24-h enzyme delivery with moderate hydration; CNC10 or ChNW10 for slower, prolonged release (optical clarity/rigidity favors CNC10; bulk hydration favors ChNW10); ChNW 5–10% for high-exudate absorption; CNC ≥ 5% for transparent, more rigid handling; biodegradation trends ChNW ≥ CNC. This map operationalizes the structure-property-function links established above.

## 3. Materials and Methods

### 3.1. Materials and Reagents

κ-Carrageenan was obtained from Sisco Research Laboratories (SRL), Maharashtra, India, and used as received. Cellulose nanocrystals (CNC) and chitin nanowhiskers (ChNW) were produced in-house from agro-industrial waste biomass using conventional acid-hydrolysis protocols. Bromelain was extracted from locally purchased fresh pineapple as detailed in Section 3.3.1 (Preparation of crude bromelain). Other reagents included glycerol, zinc chloride (ZnCl_2_), phosphate-buffered saline (PBS, pH 7.4), ethanol, Bradford reagent, and bovine serum albumin (BSA). All reagents were of analytical grade and were used without further purification. Unless otherwise stated, aqueous solutions were prepared with deionised water. Bacterial cultures of *S. aureus* and *K. pneumoniae* were obtained from the Department of Microbiology and Biotechnology, St. Thomas’ College, Pala, Kerala, India. Test organisms were maintained in peptone broth and plated on nutrient agar (HiMedia, Mumbai, India).

### 3.2. Hydrogel Fabrication

#### 3.2.1. Base κ-Carrageenan Matrix-Base Hydrogel

A 2 wt% κ-carrageenan solution was prepared in deionised water by heating and stirring at 85 ± 5 °C for 30 min. Glycerol (30 wt% relative to κ-carrageenan) was added as a plasticiser, and the mixture was mixed until a homogeneous, viscous solution was obtained. The solution was cast into Petri dishes, and ionotropic crosslinking was performed in 1 wt% ZnCl_2_ for 30 min, followed by rinsing with deionised water and storage at 4 °C.

#### 3.2.2. Cellulose Nanocrystal (CNC)–Reinforced Hydrogels

CNC was incorporated into κ-carrageenan at 5 or 10 wt% (relative to κ-carrageenan). Blends were stirred at 85 ± 5 °C for 30 min, and then glycerol (30 wt% to κ-carrageenan) was added. The mixture was cooled to 30 °C. Bromelain solution (5 mg mL^−1^) was added at a constant enzyme-to-κ-carrageenan mass ratio across all formulations. The mixture was cast and crosslinked with 1 wt% ZnCl_2_ for 30 min, rinsed with deionised water, and stored at 4 °C.

#### 3.2.3. Chitin Nanowhisker (ChNW)–Reinforced Hydrogels

ChNW was incorporated into κ-carrageenan at 5 or 10 wt% (relative to κ-carrageenan) and processed under the same conditions as CNC reinforced hydrogels. After cooling to 30 °C, bromelain solution (5 mg mL^−1^) was added, followed by casting, crosslinking (1 wt% ZnCl_2_, 30 min), rinsing with deionised water, and storage at 4 °C.

#### 3.2.4. Formulations and Coding

Five formulations were prepared in parallel under identical experimental conditions to enable direct comparison: base hydrogel (2 wt% κ-carrageenan; no nanofiller), CNC5 (5 wt% CNC with bromelain), CNC10 (10 wt% CNC with bromelain), ChNW5 (5 wt% ChNW with bromelain), and ChNW10 (10 wt% ChNW with bromelain).

### 3.3. Bromelain Loading

#### 3.3.1. Preparation of Crude Bromelain

Fresh pineapples (Ananas comosus) were washed, peeled, and the stems and cores were homogenised to obtain a pulp enriched in bromelain. The pulp was centrifuged (Rota 3RV/Fm refrigerated centrifuge; 10,000 rpm, 10 min, 4 °C) and the supernatant was treated with 80% (*v*/*v*) cold ethanol to precipitate proteins, yielding a freeze-dried crude bromelain powder after a second centrifugation and washing step.

The protein concentration was determined using the Bradford assay [45], with BSA as the standard.

#### 3.3.2. Loading into Hydrogels: Timing and Dose

Immediately before hydrogel fabrication, crude bromelain was reconstituted in PBS (pH 7.4) to 5 mg mL^−1^. For CNC5, CNC10, ChNW5 and ChNW10 formulations, 1.0 mL of the 5 mg mL^−1^ bromelain solution was added to the κ-carrageenan blends after cooling to 30 °C and immediately before casting and ionotropic crosslinking. The base hydrogel received no enzyme. All formulations were prepared in parallel under identical conditions to enable direct comparison.

### 3.4. Physicochemical Characterisation

#### 3.4.1. Hydrogel Integrity and Chemical Resilience

An initial macroscopic assessment of the hydrogels was carried out to evaluate their visual and physical characteristics, including colour, transparency, homogeneity, flexibility, and qualitative integrity. The hydrogels were cast into uniform circular moulds (11 cm diameter) and allowed to gel at ambient temperature (22 ± 2 °C). Once fully set, the samples were examined for apparent nanofiller dispersion (absence of visible aggregates), absence of phase separation or precipitation, and overall structural integrity. Texture-related features, such as brittleness, elasticity, and deformation upon handling, were recorded to assess the influence of nanofiller type (CNC or ChNW) and concentration. All formulations were photographed under identical illumination.

To further assess structural robustness, the hydrogels were subjected to a short-term chemical challenge. Samples were immersed for 2 h at 37 ± 2 °C in diluted nitric acid (HNO_3_), acetic acid, methanol, N,N-dimethylformamide (DMF), 0.1 N hydrochloric acid (HCl), 0.1 N sodium hydroxide (NaOH), chloroform, and isopropyl alcohol. Following incubation, each sample was visually inspected for gross deformation (intact/slight erosion/fragmented), apparent dissolution, color change, and loss of transparency. These observations were used to evaluate the matrix’s resilience under chemically aggressive or polar environments.

#### 3.4.2. FTIR

FTIR (PerkinElmer Spectrum IR, Waltham, MA, USA) equipped with a diamond ATR accessory was used to assess interactions among κ-carrageenan, the nanofillers, and bromelain. Samples were freeze-dried and further dried to constant mass to minimise water interference. Spectra were recorded from 4000 to 400 cm^−1^ at 4 cm^−1^ resolution with 32 scans at 22 ± 1 °C. Spectra were baseline-corrected in Spectrum IR v10.6 and area-normalised before analysis.

#### 3.4.3. FE–SEM

The surface morphology of hydrogels was examined by FE–SEM (TESCAN MAIA3 XMH) to evaluate the microstructure of the polymer network. Samples were freeze-dried, mounted on aluminium stubs, and sputter-coated with a thin Au layer to minimise surface charging. Micrographs were acquired at an accelerating voltage of 5.0 kV, with magnifications ranging from ×1000 to ×23,400; images used for quantification were acquired at a consistent magnification across groups. SEM micrographs were analysed in Fiji/ImageJ (v1.54). After scale calibration and Otsu thresholding, pores were segmented (area 0.01–10 µm^2^; circularity 0.2–1.0). The equivalent circular diameter and field porosity (%) were extracted. Per-image means were computed from ≥50 pores per field, with the image as the experimental unit (≥5 fields per sample).

#### 3.4.4. Contact Angle (Wettability)

Static water contact angle measurements were performed to evaluate surface wettability. A 5 µL droplet of deionised water was gently placed on the surface of air-dried hydrogel samples using a contact-angle goniometer. Images were captured within 3 s of deposition and left/right angles were averaged. Measurements were carried out at 32 ± 2 °C and 50 ± 10% RH, with five positions per sample (n = 3 per formulation). Lower contact angles indicate higher surface hydrophilicity.

### 3.5. Hydration and Degradation

#### 3.5.1. Swelling Behaviour (Time–Dependent)

Hydrogel specimens (2 × 2 cm; thickness measured with a calliper) were dried to constant mass at 60 °C, then immersed in PBS (pH 7.4) at 37 ± 2 °C (physiological temperature). At predefined intervals over 24 h, samples were gently blotted for 10 s on filter paper and weighed immediately. The swelling ratio (%) was calculated as [(*W_t_* − *W*_0_)/*W*_0_] × 100, where *W*_0_ is the initial dry mass and *W_t_* is the mass at time *t*. Measurements were performed in triplicate (n = 3 per formulation) and reported as mean ± SD.

#### 3.5.2. Biodegradation

Biodegradation was assessed by soil-burial testing adapted from [46]. Hydrogel specimens (2 × 2 cm) were dried to constant mass at 35 °C and buried at a 2 cm depth in non-sterilized, aerobic commercial topsoil to preserve native microbiota. Tests were conducted at 25 ± 2 °C. Soil moisture was maintained at 60 ± 5% of the water-holding capacity by periodic weighing and re-watering with deionized water. Specimens were retrieved at predefined intervals, gently rinsed, dried at 35 °C to constant mass, and mass loss (%) was calculated. Degradation (%) was calculated as [(*W_t_* − *W*_0_)/*W*_0_] × 100, where *W*_0_ is the initial dry mass and *W_t_* is the dry mass after each burial interval. All measurements were performed in triplicate (n = 3 per formulation) and expressed as mean ± SD.

### 3.6. Enzyme Release Kinetics

The release of bromelain from the nanocomposite hydrogels was evaluated using phosphate-buffered saline (PBS, pH 7.4) at 37 ± 2 °C, simulating wound-exudate conditions. Hydrogel discs (uniform geometry; cut with a sterile 6 mm punch; thickness recorded) were immersed in 10 mL of PBS and incubated under gentle orbital agitation. At predetermined time intervals (2, 3, 4, 8, 24 h), 1.0 mL aliquots were withdrawn and immediately replaced with fresh PBS to maintain sink conditions. Bromelain was quantified at 280 nm (1 cm path-length quartz cuvette) using a ultraviolet–visible (UV–Vis) spectrophotometer, with calibration in PBS across the linear range and matrix-matched blanks from bromelain-free hydrogels processed identically. Cumulative release (%) was obtained by: (i) quantifying the bromelain in each withdrawn aliquot, (ii) quantifying the bromelain remaining in the bath at each time point, (iii) summing these amounts while accounting for the dilution caused by replacing sampled volumes with fresh PBS, and (iv) normalising to the initially loaded bromelain mass. All measurements were performed in triplicate (n = 3 per formulation) and reported as mean ± SD.

### 3.7. Kinetic Analysis of Bromelain Release

The cumulative bromelain release profiles were analysed using the Higuchi and Korsmeyer–Peppas models to characterise the transport mechanism within the κ-carrageenan networks.

For the Higuchi model, the fractional release was plotted against the square root of time ($t^{1/2}$) according to Equation (1):

(1) $$M_{t}/M_{\infty }=k_{H}\;t^{1/2}$$

where $k_{H}$ (h^−1/2^) is the apparent Higuchi constant.

For the Korsmeyer–Peppas model, the logarithmic form of Equation (2) was used:

(2) $$ln\;(M_{t}/M_{\infty })=ln\;k+n\;ln\;t$$

where k is the kinetic constant and n the release exponent, indicative of the transport mechanism (Fickian when n = 0.45, anomalous when 0.45 < n < 1).

Linear regression was applied to the data points within the initial release regime (here corresponding to the entire 0–24 h interval, since $M_{t}/M_{\infty }$ ≤ 0.3 for all samples). Natural logarithms (ln) were used for the transformations. The goodness-of-fit was assessed by the coefficient of determination ($R^{2}$). All fittings were performed using GraphPad prism v8.0 with uniform weighting of experimental points.

### 3.8. Antibacterial Activity

The antibacterial activity of the hydrogel formulations was assessed using the Kirby-Bauer disc diffusion method for hydrogels [47] and the agar well diffusion method for the bromelain solution [48,49], following standard microbiological procedures. Two bacterial strains were tested: *S. aureus* (Gram–positive) and *K. pneumoniae* (Gram–negative). Inocula were prepared in peptone broth, adjusted to the 0.5 McFarland standard (1.5 × 10^8^ colony-forming units (CFU) mL^−1^), and uniformly swabbed onto Mueller-Hinton agar (MHA; 38 g L^−1^; pH 7.3) plates to ensure confluent growth. For hydrogel evaluation, sterile discs (6 mm diameter) of each formulation were aseptically placed on the agar surface. For bromelain assessment, a sterile well cutter was used to create 6 mm wells, which were then filled with enzyme solutions. Hydrogels without bromelain served as negative controls. All plates were incubated aerobically at 37 ± 2 °C for 24 h. After incubation, zones of inhibition (ZOI) were measured in millimetres with a digital calliper, averaging two perpendicular readings per zone. Results were reported as mean ± SD (n = 3 plates per condition) and interpreted by comparing ZOI values between bromelain-containing and bromelain-free hydrogels, with larger zones indicating stronger antibacterial activity.

### 3.9. Cytocompatibility Assay

Cytocompatibility was evaluated by the MTT (3-(4,5-dimethylthiazol-2-yl)-2,5-diphenyl tetrazolium bromide) assay using L929 mouse fibroblast cell lines. Cells were routinely maintained in Dulbecco’s modified Eagle’s medium supplemented with 10% fetal bovine serum and 1% penicillin-streptomycin at 37 °C in a humidified 5% CO_2_ atmosphere. For the assay, cells were seeded at 5.4 × 10^4^ cells per well in 24-well plates and incubated for 24 h to allow adherence. Sterile hydrogel samples (base and bromelain-loaded formulations) were then added and incubated for 24 h and 48 h. After exposure, MTT solution was added to each well, and the plates were incubated for 3 h. Formazan crystals were solubilised, and the resulting formazan solution was transferred to a 96-well plate for measurement. Absorbance was read at 570 nm on a microplate reader, with reagent blanks subtracted. Cell viability was calculated relative to untreated cells (negative control); the base hydrogel without bromelain served as a material control. Experiments were performed in triplicate (n = 3 per condition), and data are reported as mean ± SD.

### 3.10. Statistical Analysis

Normality was assessed with the Shapiro–Wilk test and inspection of Q-Q plots; homogeneity of variances was evaluated with Levene’s test. When assumptions were not met, the Kruskal-Wallis test with Dunn’s post hoc comparisons was used. Results are reported as mean ± SD, 95% bootstrap confidence intervals (10,000 iterations), and effect sizes. All tests were two-sided with α = 0.05; significance is denoted as * *p* < 0.05, ** *p* < 0.01, *** *p* < 0.001, and **** *p* < 0.0001.

## 4. Conclusions

κ-Carrageenan hydrogels reinforced with cellulose nanocrystals (CNC) or chitin nanowhiskers (ChNW) and loaded with bromelain were produced by a standardised, fully biobased route. Physicochemical characterisation confirmed the incorporation of nanofillers and revealed nanofiller-dictated architectures: CNC formed more compact and optically clearer networks, whereas ChNW produced more hydrated and open network structures. Both reinforcements enhanced surface wettability, although their effects on bulk hydration differed. Bromelain release was tunable, with CNC5 exhibiting the highest 24 h release. In contrast, CNC10/ChNW10 exhibited a slower release. The antibacterial ranking against *Staphylococcus aureus* and *Klebsiella pneumoniae* mirrored the release profiles, indicating a release-limited effect. All hydrogels were cytocompatible with L929 fibroblasts, with ChNW giving the highest viability. Soil burial assays showed progressive mass loss over 7 days, consistent with an environmentally responsive, transient profile. Taken together, these findings outline preliminary design guidelines for enzyme-active κ-carrageenan hydrogels rather than fully validated wound dressings. The present study did not include quantitative mechanical testing, wound-relevant cell assays (such as migration), or in vivo wound models. Therefore, the functional performance of these materials in realistic wound environments remains to be established. Future work will focus on quantifying mechanical properties and implementing advanced in vitro and in vivo models to confirm whether CNC and ChNW reinforced κ-carrageenan hydrogels can safely and effectively support bromelain-based wound-related applications.

## Figures and Tables

**Table 1:** Cumulative bromelain release (%) from κ-carrageenan hydrogels reinforced with CNC or ChNW (5 or 10 wt% relative to κ-carrageenan) at the indicated time points. Values in parentheses are the interval-specific release rates in %·h^−1^, computed between consecutive time points. Measurements in PBS (pH 7.4) at 37 ± 2 °C.

Duration (h)	Base Hydrogel	CNC5	CNC10	ChNW5	ChNW10
2	7.1 (3.6)	7.5 (3.8)	4.2 (2.1)	8.0 (4.0)	6.4 (3.2)
3	10.3 (3.2)	9.9 (2.4)	5.9 (1.7)	10.4 (2.4)	8.2 (1.8)
4	14.0 (3.7)	14.9 (5.0)	7.1 (1.2)	12.6 (2.2)	10.0 (1.8)
8	17.4 (0.9)	19.1 (1.1)	10.2 (0.8)	15.9 (0.8)	12.2 (0.6)
24	24.8 (0.5)	30.9 (0.7)	14.4 (0.3)	21.8 (0.4)	16.4 (0.3)

CNC: cellulose nanocrystals; ChNW: chitin nanowhiskers.

**Table 2:** Zone of inhibition (ZOI, mm; mean ± SD) for bromelain-loaded hydrogels against *S. aureus* and *K. pneumoniae*.

Bacteria	CNC5	CNC10	ChNW5	ChNW10	Bromelain Enzyme
*S. aureus* (+)	17.1 ± 0.1 ** (94.5%)	14.3 ± 0.4 **** (79.0%)	16.2 ± 0.3 **** (89.5%)	15.3 ± 0.4 **** (84.5%)	18.1 ± 0.2 (100%)
*K. pneumoniae* (−)	15.2 ± 0.3 **** (79.2%)	14.0 ± 0.2 **** (72.9%)	15.1 ± 0.2 **** (78.6%)	13.2 ± 0.3 **** (68.0%)	19.2 ± 0.3 (100%)

Values represent ZOI diameters (mm) and the corresponding percentages relative to free bromelain (100%). Asterisk denote significance vs. free bromelain (** *p* < 0.01, **** *p* < 0.0001).

## Data Availability

The original contributions presented in this study are included in the article. Further inquiries can be directed to the corresponding authors.

## Abbreviations

The following abbreviations are used in this manuscript:

AFM	Atomic Force Microscopy
BSA	bovine serum albumin
CNC	Cellulose nanocrystals
CNC5	κ-carrageenan/CNC hydrogel containing 5 wt% CNC
CNC10	κ-carrageenan/CNC hydrogel containing 10 wt% CNC
ChNW	Chitin nanowhiskers
ChNW5	κ-carrageenan/ChNW hydrogel containing 5 wt% ChNW
ChNW10	κ-carrageenan/ChNW hydrogel containing 10 wt% ChNW
DMF	N,N-Dimethylformamide
FTIR	Fourier-transform infrared spectroscopy
FE-SEM	Field-emission scanning electron microscopy
PBS	phosphate-buffered saline
PEG	Polyethylene Glycol
PVA	Poly(Vinyl Alcohol)
SD	standard deviation
SEM	Scanning Electron Microscopy
ZOI	Zones of inhibition
